# Angiographic Restenosis in Coronary Bifurcations Treatment with Regular Drug Eluting Stents and Dedicated Bifurcation Drug-Eluting BiOSS Stents: Analysis Based on Randomized POLBOS I and POLBOS II Studies

**DOI:** 10.1155/2020/6760205

**Published:** 2020-01-21

**Authors:** Robert J. Gil, Jacek Bil, Adam Kern, Luis A. Iñigo-Garcia, Radoslaw Formuszewicz, Slawomir Dobrzycki, Dobrin Vassilev, Roxana Mehran

**Affiliations:** ^1^Department of Invasive Cardiology, Centre of Postgraduate Medical Education, Central Clinical Hospital of the Ministry of Interior and Administration, Warsaw, Poland; ^2^Department of Cardiology and Cardiosurgery, University of Varmia and Masuria, Olsztyn, Poland; ^3^Costa del Sol Hospital, Marbella, Spain; ^4^10^th^ Clinical Military Hospital, Bydgoszcz, Poland; ^5^Department of Invasive Cardiology, Medical University in Bialystok, Bialystok, Poland; ^6^Alexandrovska University Hospital, Sofia, Bulgaria; ^7^Cardiovascular Institute, Mount Sinai Medical Center, Icahn School of Medicine at Mount Sinai, New York, NY, USA

## Abstract

**Aim:**

The marked variation in bifurcation anatomy has brought about an ongoing search for stents specifically constructed for coronary bifurcations. This study aimed to analyze the angiographic restenosis prevalence and patterns and predictors of different patterns in dedicated bifurcation BiOSS® vs. current generation drug-eluting stents implanted in coronary bifurcation lesions based on data from two clinical trials POLBOS I and II.

**Methods:**

Dedicated bifurcation BiOSS® stents were compared with drug-eluting stents (DES) in patients with stable coronary artery disease (CAD) or nonST elevation acute coronary syndrome (NSTE-ACS) (POLBOS I: paclitaxel eluting BiOSS® Expert vs. DES; POLBOS II: sirolimus eluting BiOSS® LIM vs. DES). Provisional T-stenting was the default treatment. Morphological pattern of in-stent restenosis according to the modified Mehran classification adopted for bifurcation lesions was assessed with bifurcation dedicated quantitative coronary angiographic software (CAAS 5.11, Pie Medical Imaging BV, the Netherlands).

**Results:**

In total, 445 patients (222 patients in BiOSS group and 223 patients in DES group) were included into the analysis. In BiOSS group 24 cases of angiographic restenosis (10.8%) were recorded, and in DES group—17 cases (7.6%) at 12 months follow-up (angiographic control rate at follow-up—90.3%). In the BiOSS group most frequent medina classification in restenotic cases was 0.0.1 (25%), whereas in DES—0.0.1 and 0.1.1 (23.5% each). In multivariate regression analysis proximal optimization technique was associated with the lowest chance for restenosis (OR 0.15, 95% CI 0.06–0.33), whereas diabetes on insulin was associated with the highest risk of restenosis (OR 4.21, 95% CI 1.48–11.44).

**Conclusions:**

The angiographic restenosis pattern and rate was similar between BiOSS stents and DES in coronary bifurcation lesions.

## 1. Introduction

Presently, percutaneous coronary interventions (PCI) with stent deployment are the most commonly performed procedures in the treatment of symptomatic coronary artery disease (CAD). In the last three decades, PCI with stent implantation have changed the practice of cardiology. In large trials, drug-eluting stents (DES) were associated with a significant decrease in in-stent restenosis (ISR) rates. In consequence, DES technology was swiftly and widely adopted enabling more complex interventions, also within coronary bifurcations. Nevertheless, the coronary bifurcation lesions still pose a therapeutic challenge and predispose to increased rates of periprocedural complications as well as ISR and stent thrombosis [1, 2].

We previously reported 12 months follow-up of pooled data form POLBOS I and POLBOS II trials [3]. The aim of this study is to analyze the angiographic restenosis prevalence and patterns and predictors of different patterns in dedicated bifurcation BiOSS® vs. current generation drug-eluting stents implanted in coronary bifurcation lesions.

## 2. Methods

### 2.1. Study Design

POLBOS I and POLBOS II were international, randomized, open-label, controlled trials, that have been described previously [4, 5]. The Local Ethics Committee of every participating center approved the study protocol (This trial is registered with ClinicalTrials.gov: POLBOS I—NCT02192840, POLBOS II—NCT02198300) [4, 5].

### 2.2. Interventional Procedure

After providing the written informed consent, subjects were randomized to one of two treatment strategies: BiOSS Expert® (in POLBOS I)/BiOSS LIM® (in POLBOS II) stent deployment or DES deployment [4, 5].

In both groups provisional T-stenting was the default treatment. A single stent was deployed in the main vessel—the main branch—across the side branch (SB) [6]. Bifurcation lesions were assessed visually according to Medina classification [7]. Main vessel predilatation and/or SB predilatation was done according to the operator's discretion. Then, the stent was implanted in the main vessel—the main branch. Next, the proximal optimization technique (POT) was suggested with a short noncomplaint balloon in the proximal part of the main vessel stent. After rewiring of the SB, post-dilatation and stent implantation was performed, if indicated. The procedure was completed with final kissing balloon (FKB) dilation. In BiOSS® group, it was left to the operator's decision, while in DES group, it was proceeded according to results of a second randomization.

### 2.3. Follow-Up

Clinical follow-up was performed with office visits or over telephone 1, 6, and 12 months after the procedure. Adverse events were recorded throughout the whole study period. Importantly, a follow-up coronary angiography was done at 12 months or earlier if clinically indicated.

### 2.4. Endpoints

The primary endpoint was to analyze the angiographic restenosis prevalence and patterns and predictors of different patterns in dedicated bifurcation BiOSS® vs. current generation DES implanted in coronary bifurcation lesions.

### 2.5. Angiographic Analysis

We evaluated the morphological pattern of in-stent restenosis according to the modified Mehran classification adopted for bifurcation lesions [8]. We demerged the stent into corresponding parts of the bifurcation, i.e. main vessel (MV), main branch (MB) and SB—the last one is the inseparable part of the bifurcation complex. In each modified Mehran's pattern (I–IV) we proposed subtypes to depict the restenosis location (in MV, in MB, in SB, or in combinations).

Quantitative coronary angiographic (QCA) analysis was performed using the dedicated bifurcation software CAAS 5.11 (Pie Medical Imaging BV, the Netherlands). In all cases calibration was performed with the guiding catheter. Three bifurcation segments (MV, MB, SB) were analyzed separately according to the European Bifurcation Club Consensus (EBC) [9]. Subsequent parameters were registered: reference vessel diameter (RVD), minimal lumen diameter (MLD) as well as lesion length. Percentage diameter stenosis (%DS), acute lumen gain (ALG), and late lumen loss (LLL) were computed as described previously [10]. The point of bifurcation (POB) was set out automatically by the software and defined as the mid-point of the largest circle that can be fitted in the bifurcation area, touching all 3 contours, as previously described [11].

In addition, balloon to artery ratios for both groups and subgroups were computed. This parameter was calculated as the ratio of the maximum balloon diameter, using the maximal implantation or post-dilatation pressure, and the RVD obtained before the procedure [12].

### 2.6. Statistical Analysis

Continuous variables were presented as mean ± standard deviation (SD). Categorical data were presented as numbers (%). Continuous variables were compared using an unpaired two-sided Student *t*-test, and categorical data using the *χ*^2^ test or Fisher exact test, as appropriate. If the distribution was not normal on the Shapiro–Wilk test, the Wilcoxon signed-rank and Mann–Whitney *U*-tests were used. *P* values of < 0.05 were considered statistically significant. In further analysis, univariable and multivariable logistic regression analyses were performed. A significance level was set at 0.05. Statistical analyses were performed using R 3.0.2 for OS (R Foundation, Vienna, Austria).

## 3. Results

### 3.1. General Characteristics

The study cohort included 445 patients (222 patients in BiOSS® group and 223 patients in DES group) were included into the analysis. Baseline patient characteristics with and without restenosis is shown in Table 1. In patients with restenosis in DES group higher rates of diabetes (33% vs. 52.9%) and the history of smoking (12.5% vs. 41.2%) and a lower rate of hypertension (91.7% vs. 76.5%) were observed comparing with BiOSS group. Procedural details are shown in Table 2. In patients with restenosis in DES group higher rates of side branch predilatation (29.2% vs. 47.2%), final kissing balloon technique (25% vs. 52.9%), and additional stent deployment in the side branch (20.8% vs. 47.1%) were registered comparing with BiOSS group.

### 3.2. Restenotic Cases

**Figure 1:**
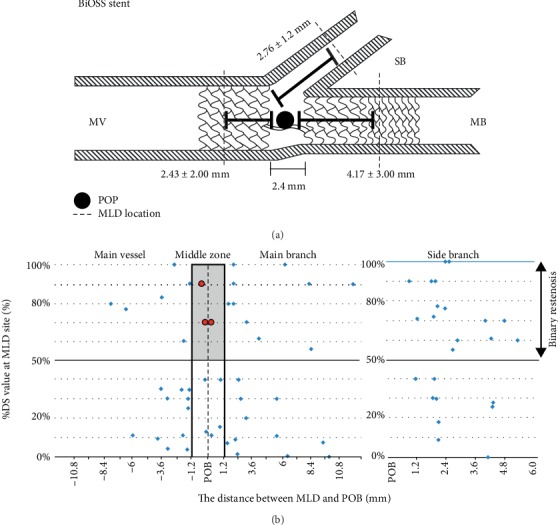
BiOSS stent – the distance between minimal lumen diameter (MLD) and the point of bifurcation (POB).

**Figure 2:**
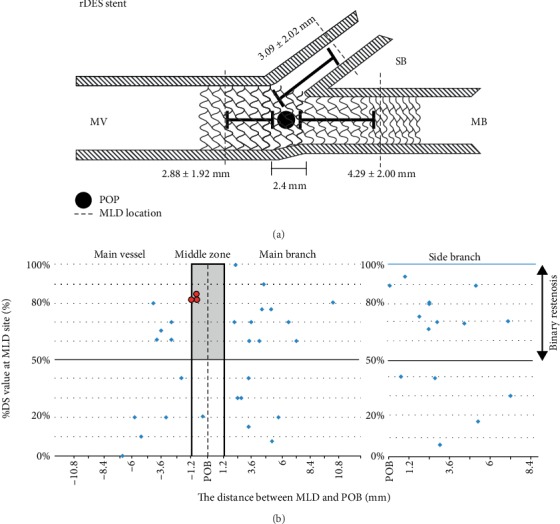
DES stent – the distance between minimal lumen diameter (MLD) and the point of bifurcation (POB).

The restenosis rate in BiOSS group was 10.80% (*n* = 24), whereas in the DES group—7.60% (*n* = 17). Localizations of restenosis were presented in Figure 1 for BiOSS and in Figure 2 for DES groups. In both groups only three cases of restenosis were found in the middle zone of the stent (at the level of carina). Restenosis rates in BiOSS group were 41.7% (*n* = 10), 37.5% (*n* = 9), and 54.2% (*n* = 13) in MV, MB, and SB, respectively. In DES group restenosis rates were as follows: 35.3% (*n* = 6), 41.2% (*n* = 7), and 64.7% (*n* = 11).

According to Medina classification true bifurcations were present in 45.8% of cases in BiOSS group, and in 52.9%—in DES group. At follow-up in restenotic cases in the BiOSS group most frequent Medina classification was 0.0.1 (25%), whereas in DES—0.0.1 and 0.1.1 (23.5% each) pattern (Supplementary Figure 1).

### 3.3. Modified Mehran Classification Adopted for Bifurcation Lesions

Restenosis type I was recorded in 58.3% in BiOSS group, whereas in DES group the rate was 52.9%. The remaining types were less common (type II: 20.8% vs. 29.4%; type III: 8.3% vs. 11.8%; type IV: 12.5% vs. 5.9%). Restenosis type IA (focal, in MV) was most frequently recorded in BiOSS group (28.6%). Interestingly, restenosis type IC (focal, in SB) was most common in DES group (17.7%) (Supplementary Tables 1 and 2).

### 3.4. Late Lumen Loss and Balloon to Artery Ratio

The change in late lumen loss in the whole population is presented in the Supplementary Figure 2. In the BiOSS group in the whole population the mean LLL in MV, MB, and SB were 0.32 ± 0.16 mm, 0.38 ± 0.18 mm, and 0.15 ± 0.07 mm, respectively, whereas in restenotic cases these values were as follows: 1.69 ± 1.22 mm, 1.30 ± 1.26 mm, and 1.05 ± 0.85 mm. In the DES group, in the whole population, the mean LLL in MV, MB, and SB were 0.24 ± 0.19 mm, 0.28 ± 0.2 mm, and 0.13 ± 0.09 mm, respectively, whereas in restenotic cases these values were as follows: 1.30 ± 1.01 mm, 1.35 ± 0.77 mm, and 0.58 ± 0.82 mm (Table 3).

The mean values of balloon to artery ratio were highest in MV in both groups, however were significantly higher in DES group than in BiOSS group (1.14 vs. 1.28) (Table 3). In other parts of bifurcation there were no significant differences in BA/A ratio between MB and SB. Moreover, mean values of balloon to artery ratio were higher in nonstenotic DES cases than in nonrestenotic BiOSS cases.

### 3.5. Left Main Subgroup

When analyzing LLL values for LM and nonLM cases it was shown that both in BiOSS and DES. LLL values were smaller in LM cases than in non-LM cases. In LM cases for BiOSS the values were: 0.21 ± 0.14 mm, 0.27 ± 0.1 mm, and 0.12 ± 0.1 mm for MV, MB, and SB and in the DES: 0.19 ± 0.13 mm, 0.23 ± 0.14 mm, and 0.14 ± 0.07 mm, respectively. Whereas for nonLM cases the values were for BiOSS: 0.37 ± 0.14 mm, 0.42 ± 0.12 mm, and 0.16 ± 0.07 mm and for DES: 0.26 ± 0.16 mm, 0.31 ± 0.14 mm, and 0.14 ± 0.08 mm, respectively (Supplementary Figure 3).

### 3.6. Predictors of ISR according to Patterns

In the multivariate analysis, proximal optimization technique (OR 0.150, 95% CI 0.061–0.327, *p* < 0.001) and age (OR 0.959, 95% CI 0.922–0.996, *p* < 0.029) were associated with reduced rate of ISR , whereas MV predilatation (OR 2.643, 95% CI 1.175–6.784, *p* = 0.028), the history of CABG (OR 2.771, 95% CI 0.991–7.100, *p* = 0.040) and DM treated with insulin (OR 4.213, 95% CI 1.483–11.444, *p* = 0.005) were associated with higher recurrence of ISR. In the regression model there was no interaction between POT and MV predilatation (*p*_interaction_ = 0.712; OR 1,545, 95% CI 0.202–32.295) (Supplementary Tables 3–5).

## 4. Discussion

In the studied population the angiographic restenosis was numerically more frequent in BiOSS group than in the DES group, however without statistical significance. The restenosis rate between BiOSS and DES was similar in case of LM lesions (2.30% vs. 2.70%), while in nonLM lesions in BiOSS group restenosis rate was almost 4-fold higher (8.50%), and in DES—almost 2-fold higher (4.90%). Therefore, it is likely that thick struts in BiOSS stent do not play such a key role in restenosis phenomenon in LM as in smaller vessels [13].

The fact that largest LLL value was located relatively far from the point of bifurcation in the MV and in the MB in BiOSS stents, confirms a thesis that mid part with only two connecting struts of the BiOSS stent is not a weak point predisposing to restenosis. Interestingly the similar profile was observed in the DES group (2.88 ± 1.92 mm and 4.29 ± 2.0 mm, respectively) (Figures 1 and 2).

The Mehran classification was evolved to characterize of ISR morphology. This classification was proved to predict the necessity for revascularization [14]. Although it was initially used in ISR in bare metal stents, this system was also proved to possess a prognostic value in ISR associated with DES [15]. Recently, we developed the modified Mehran classification adopted for coronary bifurcation lesions [8]. We did it since bifurcation lesions are more complex, and different restenosis localization might have a significant impact on the prognosis, especially in the distal left main. Type I was the type most observed, both in BiOSS and DES groups (58.3% vs. 52.9%, respectively), and the remaining types were less common (type II: 20.8% vs. 29.4%; type III: 8.3% vs. 11.8%; type IV: 12.5% vs. 5.9%) but still without significant differences. Also, the lesion location according to Medina classification at the baseline and at follow-up did not differ between BiOSS and DES groups (Supplementary Figure 1). Based on that we are allowed to say that restenosis patterns are similar for both analyzed populations (BiOSS and DES).

We know from our previous papers that thickness of the struts plays an important role in neointimal proliferation [16, 17]. Therefore, it is not surprising that in this study mean values of LLL in MV and MB were higher in BiOSS group compared to DES group with no difference for SB (rarely treated vessel, usage of small balloons). The averaged LLL value was significantly bigger only for MV in BiOSS group (Table 3). This shows a potential solution for results optimization by introducing to the market a thin-struts BiOSS version (LIM C) with a cobalt-chromium platform [18].

We showed that both in BiOSS and DES LLL values were smaller in LM cases than in nonLM cases that suggests less impact of strut thickness on neointimal proliferation and the positive role of optimization techniques (final kissing balloons and POT) more frequently used in LM treatment (Table 2).

Meticulous analysis of the LLL distribution throughout the stent showed that the LLL was significantly larger for a distal part (MB) in comparison with the proximal part (MV) or the middle part in both groups. By protocol this stent part was not optimized by additional techniques. In our opinion numerically higher values of LLL for MB suggest the need for a novel technique, analogue to POT, distal optimization technique (DOT) (Table 3).

Balloon to artery ratio being an indicator of the aggressiveness of the treatment protocol is a parameter worth further evaluation. Our analysis showed that the level of the abovementioned aggressiveness was similar for both analyzed groups, however a little more for LM subgroups. The mean values of this parameter were highest for MV in both groups, however were significantly higher in DES group than in BiOSS group (1.14 vs. 1.28) (Table 3). This might be associated with more frequent use of POT in DES group. In other parts of bifurcation there were no significant differences between MB and SB. Moreover, mean values of balloon to artery ratio were higher in nonrestenotic DES cases than in nonrestenotic BiOSS cases. This might have influenced the lower rate of restenosis in DES which was also associated with lower LLL (Table 3). It is worthy to stress that the impact of POT, especially in distal LM stenosis, was confirmed in other studies, such as the study by Kagai et al. who showed that POT might decrease rates of MACE and TLR [19].

The mean values of balloon to artery ratio were significantly higher in LM cases. Although, in the literature it was described that stent overexpansion increased the early minimum lumen diameter, but also increased the occurrence of late lumen loss at the distal edge of the stent, this was not the case in our material [20]. Also, one should stress that LLL values for MB in nonLM cases were bigger than in LM with no difference between BiOSS and DES. These findings also support already mentioned need for use of DOT to optimize the long-term outcomes.

## 5. Study Limitations

Although in line with similar studies identified in the literature, the size of the analyzed population was comparatively small. Secondly, the application of various stent types in DES group was also a limitation, however the aim of such study design was to mimic everyday clinical practice. Our new classification for restenotic patterns after coronary bifurcation stenting requires validation in the larger, prospective population.

## 6. Conclusions

Our analysis showed that despite the differences in DES and BiOSS stents structure, the vessel response did not differ significantly, especially in LM lesions. The angiographic restenosis profile was similar between BiOSS stents and DES, and the middle zone with only two struts of the BiOSS stent was not a weak point predisposing to restenosis. In regression analysis, it was shown that POT was crucial in the treatment of each bifurcation in regards to binary restenosis. Nevertheless, also the correction of distal part of the stent (DOT) seems to be required to improve the outcomes, i.e. angiographic restenosis reduction both in LM and nonLM bifurcations.

## Figures and Tables

**Table 1:** Characteristics of the study population.

	BiOSS group		DES group	
	No ISR	ISR	No ISR	ISR
	*N* = 198 (%)	*n* = 24 (%)	*N* = 206 (%)	*n* = 17 (%)
Age (years)	66.6 ± 9.7	65.2 ± 12.6	66.5 ± 9.1	65.6 ± 9.5
Men (%)	141 (71.2%)	19 (79.2)	145 (70.4%)	14 (82.4)
HTN	158 (79.8%)	22 (91.7)	158 (76.7%)	13 (76.5)^∗∗^
Hypercholesterolemia	143 (72.2%)	17 (70.8)	136 (66%)	14 (82.4)^∗^
Diabetes type 2	82 (41.4%)	8 (33.3)	63 (30.6%)	9 (52.9)^∗,∗∗^
Diabetes type 2 on insulin	19 (9.6%)	4 (16.7)	12 (5.8%)	4 (23.5)^∗^
Prior MI	85 (42.9%)	14 (58.3)^∗^	82 (39.8%)	8 (47.1)
Prior percutaneous coronary intervention	101 (51%)	11 (45.8)	109 (52.9%)	6 (35.3)^∗^
CABG	17 (8.6%)	4 (16.7)	19 (9.2%)	3 (17.6)
CKD	22 (11.1%)	1 (4.2)	16 (7.8%)	3 (17.6)
Smoking	44 (22.2%)	3 (12.5)	50 (24.3%)	7 (41.2)^∗,∗∗^
Indications for revascularization
Stable CAD	167 (84.3%)	19 (79.2)	176 (85.4%)	10 (58.8)^∗,∗∗^
NSTE-ACS	31 (15.7%)	5 (20.8)	30 (14.6%)	7 (41.2)^∗,∗∗^
Medina 1.1.1; 1.0.1; 0.1.1	167 (84.3%)	11 (45.8)^∗^	176 (85.4%)	9 (52.9)^∗^
LM bifurcation	57 (28.8%)	5 (20.8)	51 (24.8%)	6 (35.3)

^∗^ *P* < 0.05 No ISR vs. ISR in BiOSS or DES groups. ^∗∗^*P* < 0.05 ISR between BiOSS and DESs groups. CAD: coronary artery disease, CKD: chronic kidney disease; CABG: coronary artery bypass graft; ISR: in-stent restenosis; HTN: arterial hypertension; LM: left main; MI: myocardial infarction; NSTE-ACS: nonST elevation acute coronary syndrome.

**Table 2:** Periprocedural characteristics.

Parameter	BiOSS group		DES group	
	No ISR	ISR	No ISR	ISR
	*N* = 198 (%)	*n* = 24 (%)	*N* = 206 (%)	*n* = 17 (%)
MV predilatation	117 (59.1)	20 (83.3)^∗^	145 (70.4)	14 (82.4)
SB predilatation	67 (33.8)	7 (29.2)	57 (27.7)	8 (47.1)^∗,∗∗^
Nominal stent diameter [mm]	–	–	3.34 ± 0.45	3.22 ± 0.51
Nominal stent diameter in MV [mm]	3.70 ± 0.33	3.72 ± 0.41	–	–
Nominal stent diameter in MB [mm]	3.01 ± 0.34	3.00 ± 0.38	–	–
Nominal stent length [mm]	17.44 ± 1.54	18.21 ± 2.50	20.28 ± 4.32	20.53 ± 5.62
Sirolimus eluting	88 (44.4)	14 (58.3)	148 (71.8)	7 (41.2)^∗,∗∗^
Paclitaxel eluting	110 (55.6)	10 (41.7)	58 (28.2)	10 (58.8)^∗,∗∗^
POT	81 (40.9)	2 (8.3)^∗^	152 (73.8)	1 (5.9)^∗^
FKB	65 (32.8)	6 (25)	101 (49)	9 (52.9)^∗∗^
stent in SB	17 (8.6)	5 (20.8)	7 (3.4)	8 (47.1)^∗,∗∗^

^∗^ *P* < 0.05 No ISR vs. ISR in BiOSS or rDES groups. ^∗∗^*P* < 0.05 ISR between BiOSS and rDES groups. FKB: final kissing balloon; ISR: in-stent restenosis; MB: main branch; MV: main vessel; POT: proximal optimization technique; SB: side branch.

**Table 3:** Balloon to artery ratio and late lumen loss.

	BiOSS			DES		
*Balloon to artery ratio*						
BA/RD ratio	All	Restenotic	Nonrestenotic	All	Restenotic	Nonrestenotic
MV	1.14^^^	1.19^#,^^	1.13^^^	1.28^∗^^,#,^^	1.13^#,^^	1.29^∗^^,#,^^
MB	1.11^”^	1.01^”^	1.12	1.16^”^	1.05^”^	1.17^”^
SB	0.87	0.74	0.89^#^	0.86	0.72	0.87

*Late lumen loss*						
MV	0.32 ± 0.24^^^	1.69 ± 1.22	0.15 ± 0.08^#,^^	0.24 ± 0.13^∗^	1.30 ± 1.01^∗^	0.15 ± 0.06
MB	0.38 ± 0.26^”^	1.30 ± 1.26	0.27 ± 0.13^”^	0.28 ± 0.19^”^	1.35 ± 0.77	0.19 ± 0.11^∗^
SB	0.15 ± 0.05	1.05 ± 0.84	0.04 ± 0.03	0.13 ± 0.07	0.58 ± 0.82	0.09 ± 0.05

^∗^BiOSS vs. DES in corresponding subgroups. ^#^MV vs. MB, ^^^MV vs. SB, ^”^MB vs. SB.

## Data Availability

Previously reported data were used to support this study and are available at DOI 10.5603/CJ.a2017.0098. These prior studies (and datasets) are cited at relevant places within the text as references [4, 5].

## Supplementary Materials

Supplemental Materials Supplementary Figure 1: medina classification change in restenotic cases baseline and at follow-up in BiOSS and DES groups. Supplementary Figure 2: late lumen loss change along the stent in BiOSS and DES groups. Supplementary Figure 3: (a) late lumen loss in LM and nonLM subgroups. (b) Balloon:artery ratio in LM and nonLM subgroups. Supplementary Table 1: BiOSS: Modified Mehran's classification adapted for restenosis in bifurcation lesions. Supplementary Table 2: DES: Modified Mehran's classification adapted for restenosis in bifurcation lesions. Supplementary Table 3: logistic regression for binary restenosis in the whole population. Supplementary Table 4: logistic regression for binary restenosis in the LM population. Supplemenatry Table 5: logistic regression for binary restenosis in the nonLM population.
