# Supplementary material for: Angiographic Restenosis in Coronary Bifurcations Treatment with Regular Drug Eluting Stents and Dedicated Bifurcation Drug-Eluting BiOSS Stents: Analysis Based on Randomized POLBOS I and POLBOS II Studies
Source: Cardiovasc Ther. 2020 Jan 21;2020:6760205. doi: 10.1155/2020/6760205 (PMC7204374; doi:10.1155/2020/6760205)
Supplement: Supplemental Materials — Supplementary Figure 1: medina classification change in restenotic cases baseline and at follow-up in BiOSS and DES groups. Supplementary Figure 2: late lumen loss change along the stent in BiOSS and DES groups. Supplementary Figure 3: (a) late lumen loss in LM and nonLM subgroups. (b) Balloon:artery ratio in LM and nonLM subgroups. Supplementary Table 1: BiOSS: Modified Mehran's classification adapted for restenosis in bifurcation lesions. Supplementary Table 2: DES: Modified Mehran's classification adapted for restenosis in bifurcation lesions. Supplementary Table 3: logistic regression for binary restenosis in the whole population. Supplementary Table 4: logistic regression for binary restenosis in the LM population. Supplemenatry Table 5: logistic regression for binary restenosis in the nonLM population. [file 6760205.f1.pdf]

Supplementary materials

Suppl Tables 1 – 5: appropriate references in the maintext

Suppl Figures 1 – 3: appropriate references in the maintext

Suppl Figure 1. Medina classification change in restenotic cases baseline and at follow-up in BiOSS and DES groups.

Suppl Figure 2. Late lumen loss change along the stent in BiOSS and DES groups.

Suppl Figure 3. (A) Late lumen loss in LM and non-LM subgroups. (B) Balloon:artery ratio in LM and non-LM subgroups.

Supplementary Table 1. BiOSS: Modified Mehran's classification adapted for restenosis in bifurcation lesions.

Type of restenosis	Non-LM<br>N = 19	LM<br>n = 5 (%)
**I (focal restenosis, < 10 mm in stent)**	**12**	**2**
A (in MV-MB)	5	1
B (in SB)	4	1
C (both)	3	0
**II (> 10 mm within the stent)**	**4**	**1**
A (in MV-MB)	2	0
B (in SB)	0	0
C (both)	2	1
**III (> 10 mm + outside the stent)**	**1**	**1**
A (in MV-MB)	0	1
B (in SB)	0	0
C (both)	1	0
**IV (total occlusion)**	**2**	**1**
A (in MV)	0	0
B (in MB)	1	0
C (in SB)	0	1
D (in MB and SB)	1	0

Supplementary Table 2. DES: Modified Mehran's classification adapted for restenosis in bifurcation lesions.

Type of restenosis	LM<br>N = 6	Non-LM<br>n = 11
**I (focal restenosis, < 10 mm in stent)**	**2**	**7**
A (in MV-MB)	1	2
B (in SB)	1	2
C (both)	0	3
**II (> 10 mm within the stent)**	**3**	**2**
A (in MV-MB)	1	0
B (in SB)	0	0
C (both)	2	2
**III (> 10 mm + outside the stent)**	**1**	**1**
A (in MV-MB)	0	1
B (in SB)	0	0
C (both)	1	0
**IV (total occlusion)**	**0**	**1**
A (in MV)	0	0
B (in MB)	0	1
C (in SB)	0	0
D (in MB and SB)	0	0

Supplementary Table 3. Logistic regression for binary restenosis in the whole population.

Variate	Univariate analysis OR (95% CI)	p-value	Multivariate analysis OR (95% CI)	p-value
Group: BiOSS vs DES	1.380 (0.730 - 2.655)	0.325		
Group: BiOSS.Expert vs DES	1.504 (0.709 - 3.134)	0.277		
Group: BiOSS.LIM vs DES	1.238 (0.531 - 2.739)	0.606		
Sex: female vs male	0.568 (0.239 - 1.206)	0.166		
Age [increase per 1 year]	0.990 (0.957 - 1.024)	0.547	0.959 (0.922 - 0.996)	0.029
NSTEMI/UA	2.243 (1.054 - 4.525)	0.029		
arterial hypertension	1.377 (0.625 - 3.476)	0.459		
diabetes	1.335 (0.687 - 2.540)	0.383		
diabetes on insulin	2.824 (1.136 - 6.384)	0.017	4.213 (1.483 - 11.444)	0.005
Dyslipidemia	1.253 (0.627 - 2.681)	0.540		
prior myocardial infarction	1.259 (0.661 - 2.384)	0.479		
prior PCI	0.861 (0.453 - 1.629)	0.644		
prior coronary artery bypass graft	2.039 (1.785 - 4.686)	0.043	2.771 (0.991 - 7.100)	0.040
chronic kidney disease	1.011 (0.292 - 2.691)	0.984		
smoking	1.027 (0.464 - 2.098)	0.944		
Ture Bifurcation	2.280 (0.883 - 7.772)	0.127		
left main bifurcation	1.032 (0.515 - 2.213)	0.932		
main vessel predilatation	2.720 (1.248 - 6.822)	0.019	2.643 (1.175 - 6.784)	0.028
side branch predilatation	1.250 (0.629 - 2.403)	0.511		
side branch stenting	1.180 (0.339 - 3.171)	0.766		
final kissing balloon	0.627 (0.307 - 1.218)	0.181		
proximal optimization technique	0.181 (0.076 - 0.381)	<0.001	0.150 (0.061 - 0.327)	<0.001
Implantation pressure > 12 atm	1.235 (0.765 - 2.321)	0.589		
BA/RD ratio	1.324 (0.897 - 1.788)	0.856		
ALG in MV	0.566 (0.312 - 1.499)	0.331		
ALG in MB	0.761 (0.440 - 2.122)	0.518		
ALG in SB	0.898 (0.650 - 1.231)	0.632		
LM ostium coverage	1.070 (0.381 - 2.447)	0.821		
MV predilatation if %DS≥70%	2.120 (1.478 - 5.214)	0.003	2.221 (1.369 - 5.897)	0.031

PCI – percutaneous coronary intervention, UA/NSTEMI – unstable angina/non-ST-elevation myocardial infarction.

Supplementary Table 4. Logistic regression for binary restenosis in the LM population.

**Variate**	**Univariate analysis OR (95% CI)**	**p-value**	**Multivariate analysis OR (95% CI)**	**p-value**
Group: BiOSS vs DES	1.110 (0.760 - 1.445)	0.234		
Group: BiOSS.Expert vs DES	1.326 (0.891 - 2.341)	0.277		
Group: BiOSS.LIM vs DES	1.131 (0.791 - 1.872)	0.606		
Sex: female vs male	0.744 (0.349 - 1.123)	0.166		
Age [increase per 1 year]	1.320 (0.788 - 1.454)	0.547		
NSTEMI/UA	1.543 (0.875 - 2.545)	0.029		
arterial hypertension	1.231 (0.532 - 2.111)	0.459		
diabetes	1.563 (0.322 - 4.170)	0.383		
diabetes on insulin	2.110 (0.846 - 7.214)	0.017		
Dyslipidemia	1.111 (0.722 - 1.381)	0.540		
prior myocardial infarction	1.542 (0.769 - 1.733)	0.479		
prior PCI	1.121 (0.633 - 1.522)	0.644		
prior coronary artery bypass graft	1.349 (0.435 - 2.136)	0.113		
chronic kidney disease	1.511 (0.642 - 2.111)	0.984		
smoking	1.111 (0.322 - 1.908)	0.944		
Ture Bifurcation	2.122 (0.783 – 9.111)	0.127		
main vessel predilatation	2.321 (1.548 - 4.231)	0.019	2.743 (1.321 - 5.432)	0.028
side branch predilatation	1.210 (0.459 - 2.127)	0.511		
side branch stenting	1.590 (0.874 - 4.234)	0.766		
final kissing balloon	0.875 (0.643 - 0.933)	0.040	0.734 (0.548 - 0.903)	0.004
proximal optimization technique	0.341 (0.105 - 0.492)	<0.001	0.234 (0.109 - 0.392)	<0.001
Implantation pressure > 12 atm	1.235 (0.765 - 2.321)	0.589		
BA/RD ratio	1.345 (0.902 - 1.448)	0.856		
LM ostium coverage	1.170 (0.441 - 1.467)	0.821		
MV predilatation if %DS≥70%	1.895 (1.235 - 2.764)	0.002	2.011 (1.577 - 3.129)	<0.001

PCI – percutaneous coronary intervention, UA/NSTEMI – unstable angina/non-ST-elevation myocardial infarction.

Supplemenatry Table 5. Logistic regression for binary restenosis in the non-LM population.

Variate	Univariate analysis OR (95% CI)	p-value	Multivariate analysis OR (95% CI)	p-value
Group: BiOSS vs DES	1.481 (0.521 - 1.955)	0.325		
Group: BiOSS.Expert vs DES	1.604 (0.939 - 3.344)	0.277		
Group: BiOSS.LIM vs DES	1.428 (0.641 - 2.219)	0.606		
Sex: female vs male	0.782 (0.459 - 1.561)	0.166		
Age [increase per 1 year]	1.090 (0.911 - 1.132)	0.547		
NSTEMI/UA	2.010 (0.934 - 3.125)	0.209		
arterial hypertension	1.377 (0.625 - 3.476)	0.459		
diabetes	1.335 (0.687 - 2.540)	0.383		
diabetes on insulin	1.832 (1.236 - 4.121)	0.003	2.111 (1.328 - 7.135)	0.028
Dyslipidemia	1.245 (0.721 - 1.671)	0.540		
prior myocardial infarction	1.112 (0.431 – 2.103)	0.479		
prior PCI	0.922 (0.763 - 1.109)	0.644		
prior coronary artery bypass graft	3.652 (1.112 - 4.686)	0.053	2.921 (1.052 - 4.221)	0.030
chronic kidney disease	1.212 (0.312 – 2.721)	0.984		
smoking	1.111 (0.644 – 1.973)	0.944		
Ture Bifurcation	1.891 (0.723 - 6.345)	0.127		
main vessel predilatation	2.120 (1.568 - 5.441)	0.020	2.831 (1.455 - 6.111)	0.021
side branch predilatation	1.010 (0.282 - 1.813)	0.511		
side branch stenting	1.231 (0.439 - 2.211)	0.766		
final kissing balloon	0.711 (0.417 - 1.338)	0.181		
proximal optimization technique	0.231 (0.091 - 0.342)	<0.001	0.183 (0.073 - 0.427)	<0.001
Implantation pressure > 12 atm	1.341 (0.821 – 1.901)	0.589		
BA/RD ratio	1.114 (0.492 - 1.448)	0.856		
MV predilatation if %DS≥70%	1.821 (1.279 – 3.431)	0.002	2.001 (1.477 - 4.011)	0.025

PCI – percutaneous coronary intervention, UA/NSTEMI – unstable angina/non-ST-elevation myocardial infarction.

Suppl Figure 1

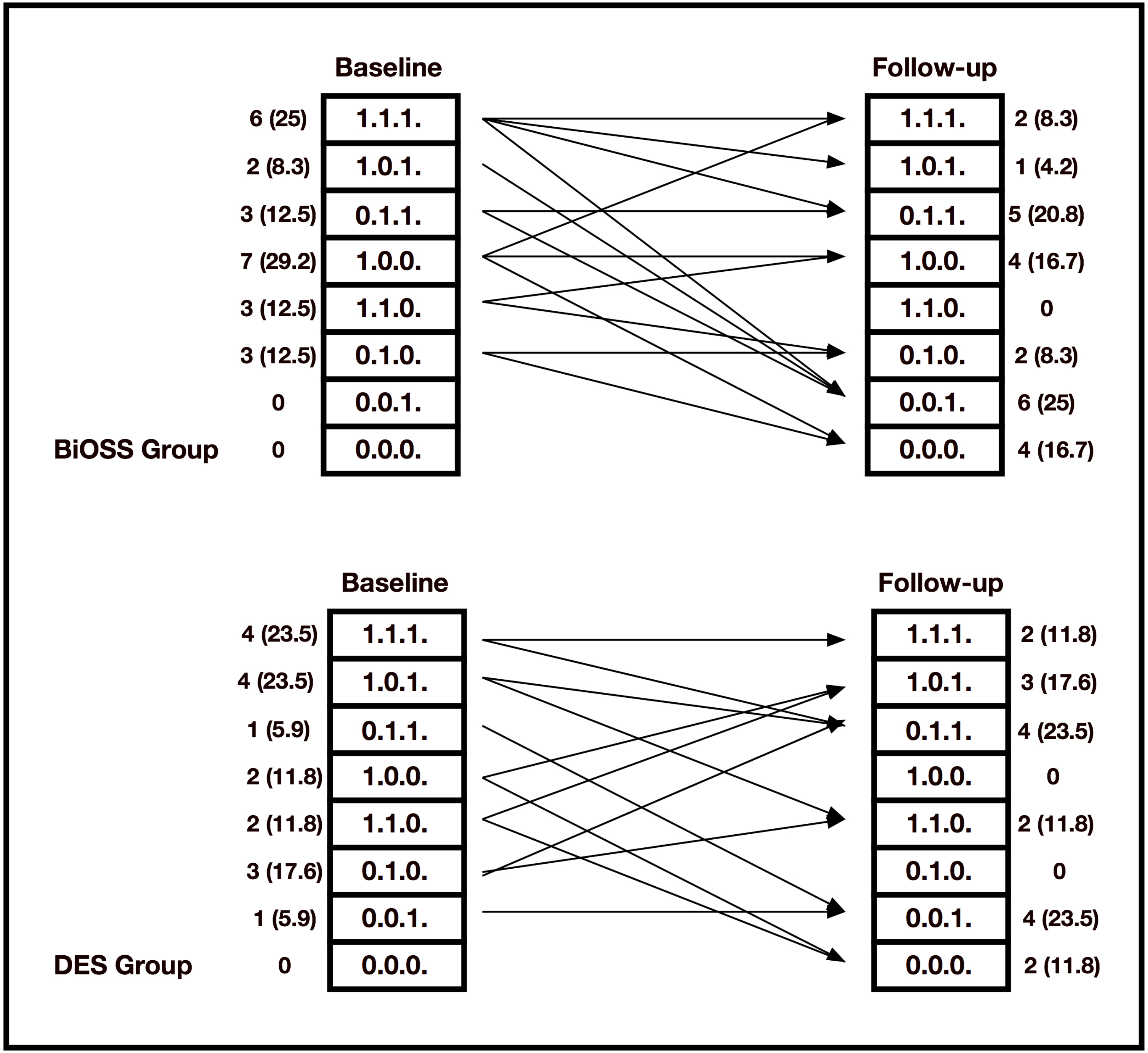

Suppl Figure
2

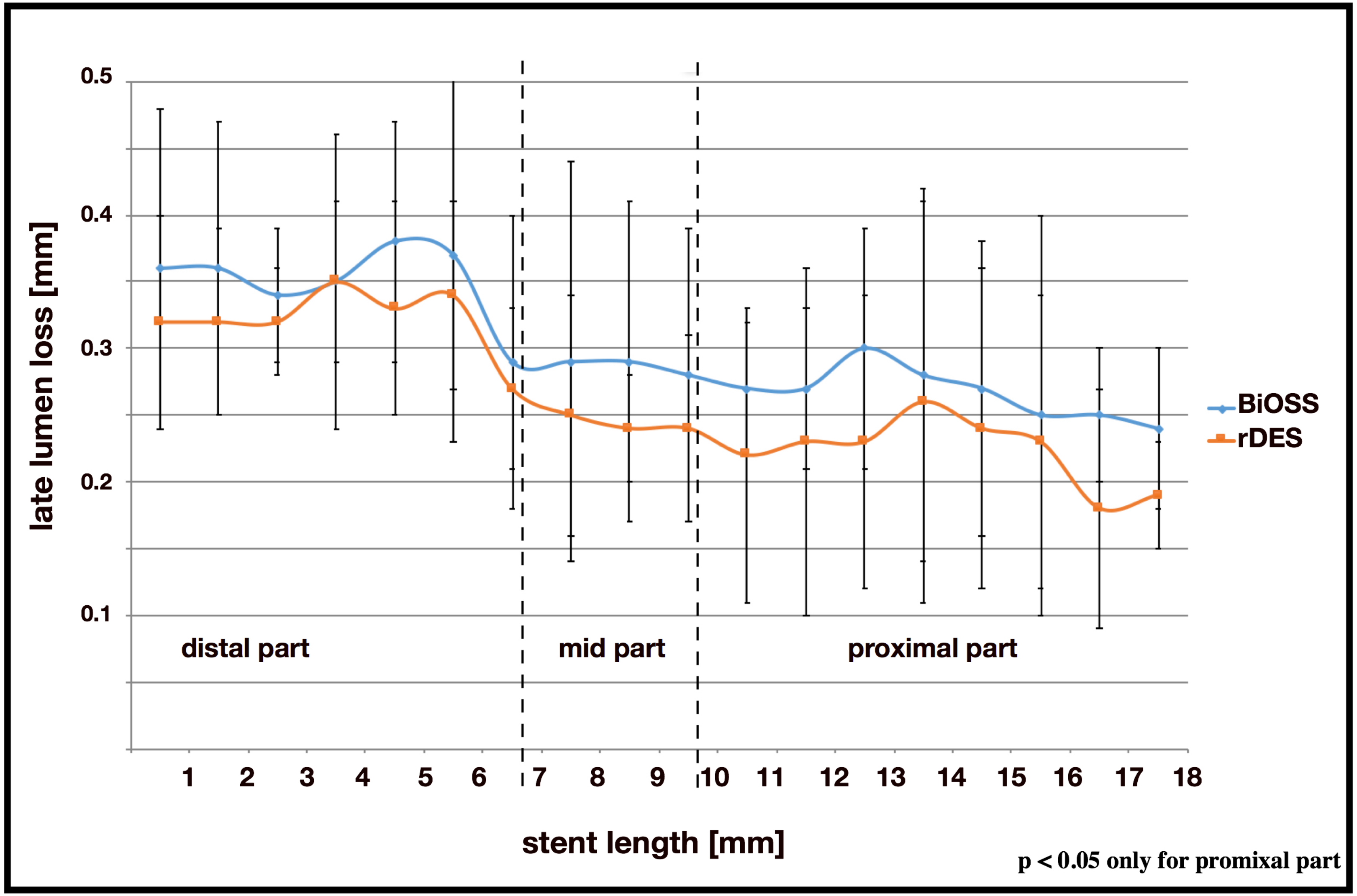

Suppl Figure 3

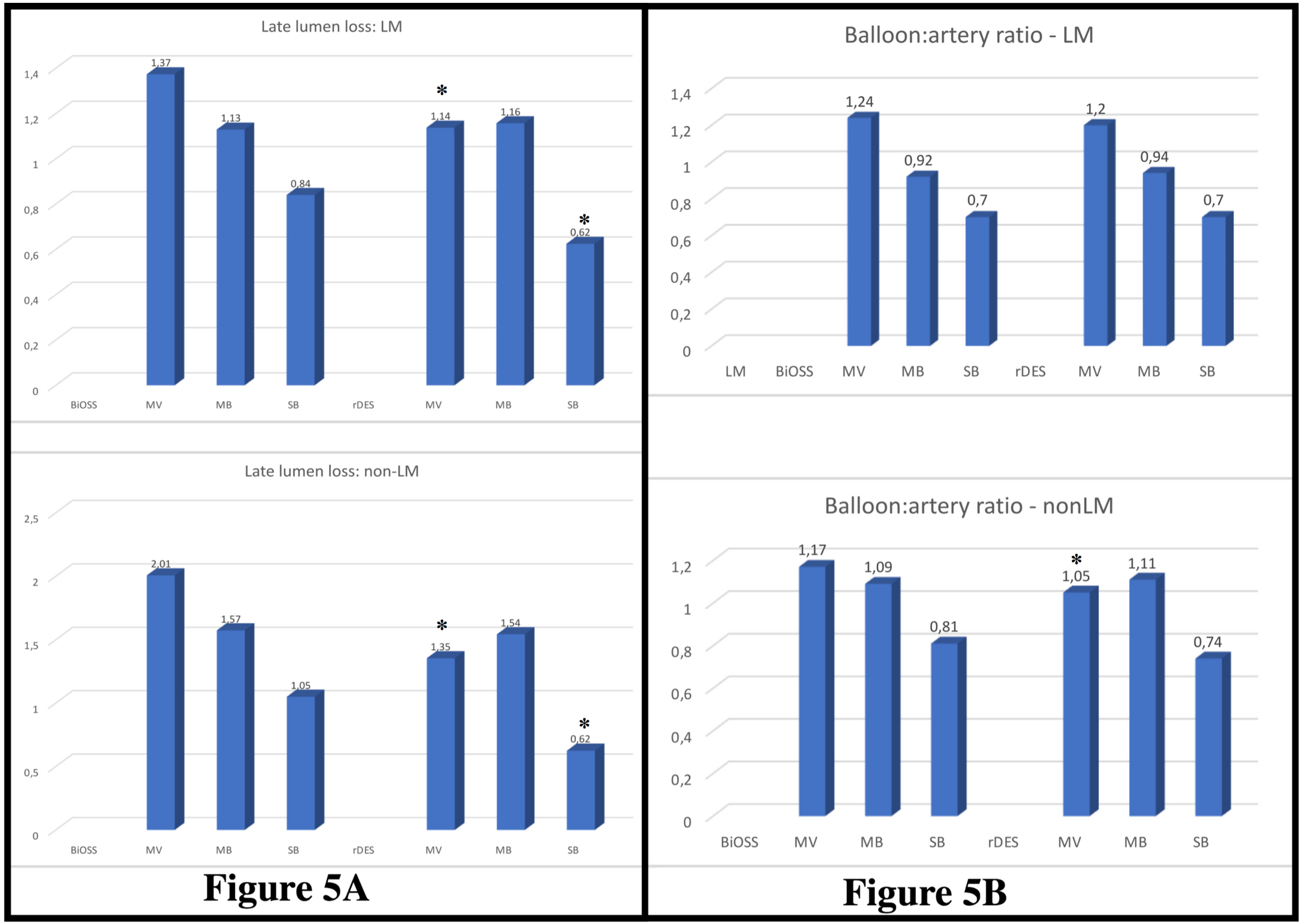

Figure 5A **Figure 5B**

***p < 0.05 for BiOSS vs DES**
